# Peer-Led Community-Based Support Services and HIV Treatment Outcomes Among People Living With HIV in Wuxi, China: Propensity Score–Matched Analysis of Surveillance Data From 2006 to 2021

**DOI:** 10.2196/43635

**Published:** 2023-03-24

**Authors:** Xiaojun Meng, Hanlu Yin, Wenjuan Ma, Jing Gu, Zhen Lu, Thomas Fitzpatrick, Huachun Zou

**Affiliations:** 1 Wuxi Municipal Center for Disease Control and Prevention Wuxi China; 2 School of Public Health (Shenzhen), Sun Yat-sen University Shenzhen China; 3 Division of Allergy and Infectious Diseases, University of Washington Seattle, WA United States; 4 Kirby Institute, University of New South Wales Sydney Australia

**Keywords:** people living with HIV, community health workers, community-based organizations, peer-led services, HIV treatment outcomes

## Abstract

**Background:**

Community-based organizations deliver peer-led support services to people living with HIV. Systematic reviews have found that peer-led community-based support services can improve HIV treatment outcomes; however, few studies have been implemented to evaluate its impact on mortality using long-term follow-up data.

**Objective:**

We aimed to evaluate the associations between the receipt of peer-led community-based support services and HIV treatment outcomes and survival among people living with HIV in Wuxi, China.

**Methods:**

We performed a propensity score–matched retrospective cohort study using data collected from the Chinese National HIV/AIDS Comprehensive Information Management System for people living with HIV in Wuxi, China, between 2006 and 2021. People living with HIV who received adjunctive peer-led community-based support for at least 6 months from a local community-based organization (exposure group) were matched to people living with HIV who only received routine clinic-based HIV care (control group). We compared the differences in HIV treatment outcomes and survival between these 2 groups using Kaplan-Meier curves. We used competing risk and Cox proportional hazards models to assess correlates of AIDS-related mortality (ARM) and all-cause mortality. We reported adjusted subdistribution hazard ratio and adjusted hazard ratio with 95% CIs.

**Results:**

A total of 860 people living with HIV were included (430 in the exposure group and 430 in the control group). The exposure group was more likely to adhere to antiretroviral therapy (ART; 396/430, 92.1% vs 360/430, 83.7%; *P*<.001), remain retained in care 12 months after ART initiation (402/430, 93.5% vs 327/430, 76.1%; *P*<.001), and achieve viral suppression 9 to 24 months after ART initiation (357/381, 93.7% vs 217/243, 89.3%; *P*=.048) than the control group. The exposure group had significantly lower ARM (1.8 vs 7.0 per 1000 person-years; *P=*.01) and all-cause mortality (2.3 vs 9.3 per 1000 person-years; *P=*.002) and significantly higher cumulative survival rates (*P=*.003). The exposure group had a 72% reduction in ARM (adjusted subdistribution hazard ratio 0.28, 95% CI 0.09-0.95) and a 70% reduction in all-cause mortality (adjusted hazard ratio 0.30, 95% CI 0.11-0.82). The nonrandomized retrospective nature of our analysis prevents us from determining whether peer-led community-based support caused the observed differences in HIV treatment outcomes and survival between the exposure and control groups.

**Conclusions:**

The receipt of peer-led community-based support services correlated with significantly improved HIV treatment outcomes and survival among people living with HIV in a middle-income country in Asia. The 15-year follow-up period in this study allowed us to identify associations with survival not previously reported in the literature. Future interventional trials are needed to confirm these findings.

## Introduction

### Survival Improved Among People Living With HIV

According to the 2020 global AIDS report, 38 million people are living with HIV globally, of whom 25.4 million are receiving antiretroviral therapy (ART) [1]. If the *95-95-95* targets updated by the Joint United Nations Program on HIV/AIDS in December 2020 are to be achieved by 2025 [2], nearly 86% of all people living with HIV worldwide will need to be initiated on ART and achieve viral suppression. China has achieved considerable success in optimizing the HIV care continuum in recent decades. Currently, an estimated 83.4% of all people living with HIV in China are receiving ART, of whom 94.2% have achieved viral suppression [3].

The overall life expectancy of people living with HIV with good adherence to ART is approaching that of the general population in many high-income countries [4,5]; however, significant differences in survival persist in resource-limited settings [6]. The use of ART to improve survival among people living with HIV depends on early initiation of treatment, adherence to ART, and retention in care [7-9]. AIDS-related mortality (ARM) remains high in certain contexts, particularly because of advanced immunosuppression at the time of ART initiation [10,11]. In addition, an increasing proportion of deaths among people living with HIV are attributed to non-ARM (NARM) [12]. A recent global systematic review showed many people living with HIV died of cardiovascular disease, non-AIDS malignancies, and liver disease [13]. To improve health outcomes among people living with HIV, effective interventions are needed to reduce both ARM and NARM.

### Community Health Workers Promote the Quality of HIV Care Among People Living With HIV

Task shifting involves the rational redistribution of tasks from the professional health workforce to community-based organizations (CBOs) and community health workers (CHWs) and is recognized by the World Health Organization (WHO) as an important strategy to optimize HIV care [14]. CHWs can play a valuable role in engaging and retaining people living with HIV in care [15,16]. CHWs strengthen the quality of HIV services as well as promote dignity and improve the quality of life for people living with HIV enrolled in those services [14]. Trials in several low-resource settings found CHWs can reduce the rates of virologic failure on ART and loss to follow-up [16-18]. A cluster randomized controlled trial in Zimbabwe found using CHWs to implement a community-based differentiated service delivery model that adapted HIV services to the preferences and expectations of adolescents significantly improved HIV viral suppression [19]. The WHO recommends community-based interventions to support ART adherence and retention in care while acknowledging that the evidence to support this recommendation is weak. Most previous evaluations of peer-led community-based interventions for people living with HIV have been conducted in high-income countries or sub-Saharan Africa, and few have reported survival as an outcome.

CBOs and CHWs have played a critical role in optimizing the HIV care continuum in China, especially in efforts to increase access to ART and improve retention in care [20]. The Chinese government has implemented a nationwide task-shifting strategy for HIV services, supporting the scale-up of CHWs through the China AIDS Fund for CBOs. Few previous studies have evaluated the effect of peer support delivered by CHWs on HIV treatment outcomes in China. We performed a propensity score–matched analysis of longitudinal data extracted from a database of HIV treatment outcomes in China to evaluate the impact of a multicomponent peer support model on ART adherence, viral suppression, and survival among people living with HIV in Wuxi, China.

## Methods

### Study Design

Using routine surveillance records on people living with HIV, we conducted a propensity score–matched cohort study of individuals who were served by local CBOs on HIV care. Convenient sampling method was used to enroll people living with HIV who had received peer-led community-based support services from local CBOs in the exposure group. In the meantime, we used propensity score matching (PSM) to enroll a comparable control group from other people living with HIV who had never received such a service.

### Study Setting

Wuxi is a city of 8.5 million people in China’s Jiangsu Province. As of December 2021, a total of 4111 people living with HIV were registered as living in Wuxi. Rainbow Family is currently the only CBO registered with the local government to provide services for people living with HIV in Wuxi. This CBO has been providing support services to people living with HIV since June 2006.

### Standard of Care for People Living With HIV in China

In 2003, China implemented free ART for people living with HIV [21]. Under this policy, all people living with HIV in China can obtain free HIV care and ART from health care providers at infectious diseases hospitals or clinics affiliated with local centers for disease control and prevention (CDCs). National guidelines suggest routine follow-up services should be delivered at 0.5, 1, 2, 3, 6, 9, and 12 months after ART initiation to monitor for adverse drug effects and adjust ART as necessary. At 13 months after ART initiation, follow-up visits are recommended every 3 months, with CD4 and viral load testing performed at least every 12 months [22].

### Peer-Led Community-Based Support Services Provided by Rainbow Family

In Wuxi, all newly diagnosed people living with HIV are informed of their diagnosis and receive their initial HIV care visit at a local CDC clinic or infectious diseases hospital. At this initial visit, health care providers will introduce people living with HIV to the peer-led community-based support services offered at Rainbow Family and provide a referral if desired. A CHW from Rainbow Family will be assigned to any referred person living with HIV in Wuxi. Not all newly diagnosed people living with HIV opt to use the peer support services provided by CBOs. Factors influencing decision-making include, but are not limited to, self-acceptance of HIV status, willingness to expose their sexual orientation to others in the CBOs, and willingness to be followed up by peer CHWs in daily life.

CHWs at Rainbow Family are people living with HIV themselves and recruited as volunteers working for CBOs in their spare time who have documented adherence to ART for at least 12 months and demonstrated strong interpersonal and communication skills. Before providing services to clients, CHWs at Rainbow Family receive education on HIV pathogenesis, prevention, and treatment, as well as training on how to provide psychological support and referrals to other medical services. CHW trainings are led by professional health care workers, including public health practitioners from the Wuxi municipal CDC, HIV doctors and nurses, mental health counselors, and experienced peer educators. After passing the professional training, the CHWs will be assigned clients who are people living with HIV and initiate the peer-led community-based support according to guidelines.

The peer-led community-based support model provided by Rainbow Family was designed as a patient-centered peer support model. CHWs build trust and rapport with people living with HIV through regular face-to-face communication, contact through WeChat (an instant messaging software widely used in China), telephone calls, home visits, and group activities. These regular interactions allow CHWs to provide health care services and support to people living with HIV that are tailored to the individual needs of clients.

**Figure 1:**
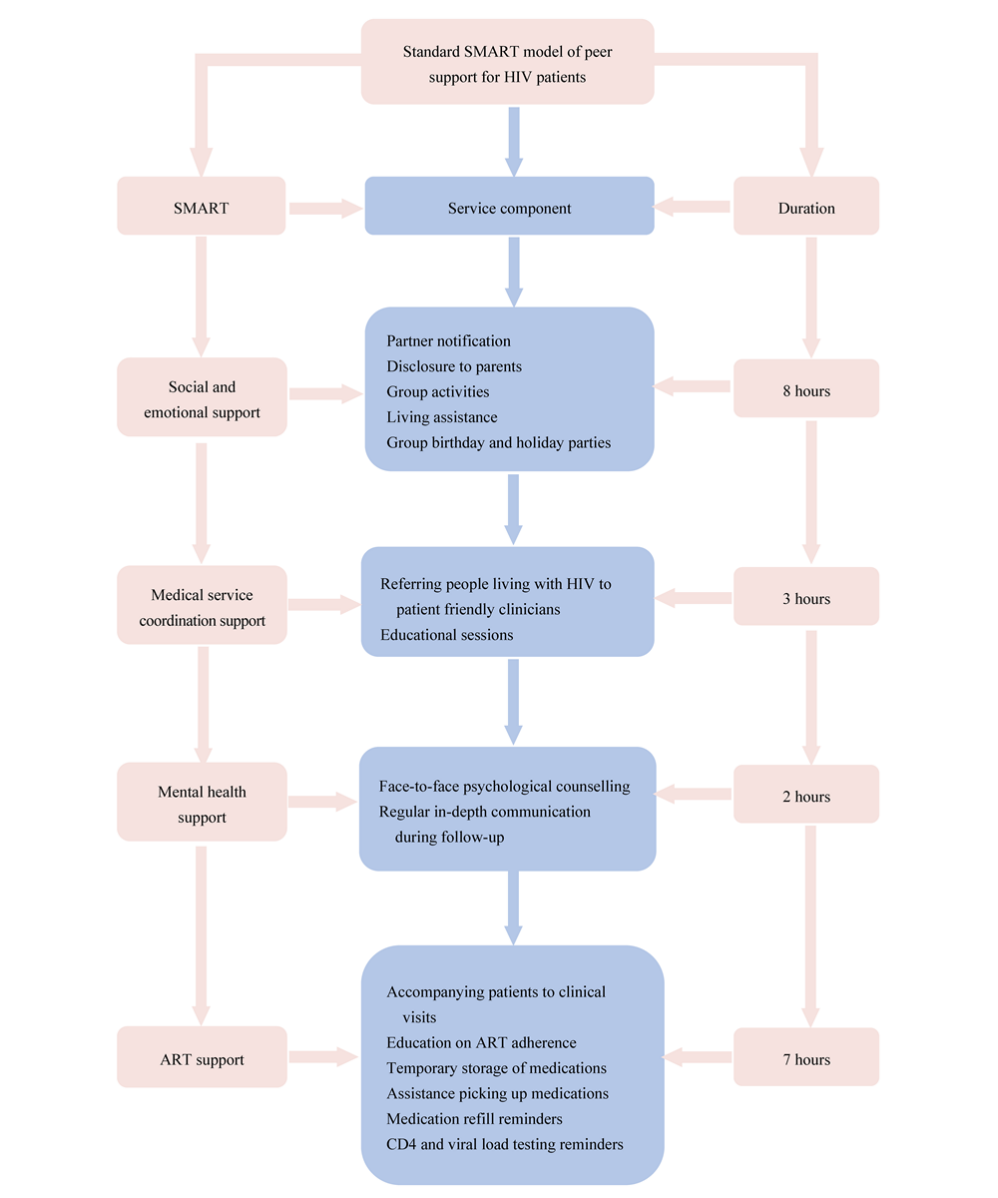
The social, medical, and mental antiretroviral therapy (SMART) model of community-based peer-led support for people living with HIV in Wuxi, China. ART: antiretroviral therapy.

A social, medical, and mental ART support (SMART) model is used as a conceptual framework to guide this service (Figure 1). This model emphasizes four forms of support: (1) social and emotional support to navigate relationships with sex partners, spouses, family members, and friends, particularly assisting people living with HIV in coping with the emotional stress associated with partner notification and disclosure of HIV status; (2) medical service coordination support to overcome HIV-related stigma and discrimination from health care providers by referring people living with HIV to clinicians with experience providing nondiscriminatory care; (3) mental health support to connect people living with HIV with in-person psychological counseling to address concurrent anxiety, depression, or other mental health disorders; and (4) ART support to provide people living with HIV with additional education emphasizing the importance of ART adherence, accompanying patients to clinic visits to help them navigate ART initiation and maintenance, and providing reminders regarding medication refills and laboratory testing to overcome treatment fatigue.

### Data Collection

In accordance with national HIV treatment guidelines, HIV diagnoses are reported via the Chinese National HIV/AIDS Comprehensive Information Management System (CNHCIMS) within 24 hours. Information on sociodemographic characteristics, risk behaviors, CD4 count, HIV viral load, and mortality is recorded in the CNHCIMS. We extracted case report data from the CNHCIMS on sociodemographic characteristics and risk factors for HIV transmission for all people living with HIV in Wuxi between January 1, 2006, and December 31, 2019. We also extracted follow-up data detailing care retention, ART adherence, CD4 and viral load monitoring, mortality, and the cause of death for the same people living with HIV between January 1, 2006, and December 31, 2021.

### Exposure Group

Rainbow Family collects and records the name and government-issued ID card number of all people living with HIV who have been assigned a CHW and received peer-led community-based support services. We obtained a list of people living with HIV who ever received peer-led community-based support from Rainbow Family, and persons on this list were linked to the CNHCIMS data by their unique government-issued ID card number. People living with HIV were assigned to the exposure group if they met the following inclusion criteria: (1) age ≥18 years at HIV diagnosis; (2) initiated ART on and before December 31, 2019; and (3) received peer-led community-based support from Rainbow Family for at least 6 months.

### Control Group

People living with HIV in Wuxi identified in the CNHCIMS who had not received peer-led community-based support from Rainbow Family were eligible to be assigned to the control group in our analysis. To adjust for potential confounders, PSM was used to match people living with HIV in the exposure group with controls. Sex, age at diagnosis, ethnicity, education, marital status, occupation, the route of HIV infection, and baseline CD4 count are factors found to be significantly associated with survival among people living with HIV in previous studies and therefore were included in our PSM model [12,23,24]. In addition, before June 2016, people living with HIV in China were only eligible to initiate ART if their CD4 count was <500 cells/μL; therefore, the year of HIV diagnosis was also included in our PSM model.

When implementing PSM, propensity scores were generated using logistic regression, with the receipt versus nonreceipt of peer-led community-based support as the dependent variable. Propensity scores were generated for each of the imputed data sets, and an average propensity score for each observation was calculated across data sets. Each patient who received peer-led community-based support services (exposure group) was matched 1:1 with a patient who received routine support (control group) according to their propensity scores, using the nearest neighbor matching method without replacement.

### Study Variables

Two sets of variables were extracted from the CNHCIMS. The first set was variables in HIV case report data set evaluated at diagnosis, which involved sex (male and female), age at diagnosis (continuous), ethnicity (Han and others), education (primary or less, secondary, and tertiary), marital status (unmarried, married, and divorced or widowed), occupation (student and others), the route of HIV infection (men who have sex with men and others including heterosexual behavior, drug use, and blood donation), the date of HIV diagnosis, and baseline CD4 count (cells/μL). All these variables were categorized into 2 or 3 subgroups where necessary and applicable. The other set was variables in the follow-up data set recorded after HIV diagnosis, in which drug collection, ART interruption, and scheduled follow-up visit were used to measure adherence to ART, and CD4 and viral load were used to measure retention in care.

### Outcomes

All data on study outcomes were extracted from the CNHCIMS, and all outcomes were assessed using these extracted data. Adherence to ART was defined as never having a documented period of ART interruption and never having a period when a scheduled follow-up clinic visit was missed >3 months. Retention in care was defined as having received either CD4 or viral load monitoring on at least 1 occasion ≥12 months after ART initiation. People living with HIV who were on ART for at least 6 months and had a documented HIV viral load ≤1000 copies per mL were considered to be virally suppressed [25].

Information on the cause of death was obtained from diagnosis codes in the CNHCIMS. Causes of death were classified into categories of ARM, NARM, and all-cause mortality according to the coding causes of death in HIV Project protocol [26]. A death was classified as AIDS-related if the cause of death was an AIDS-defining condition. All other deaths were classified as non–AIDS-related. Non–AIDS-related deaths were grouped into several subcategories based on diagnosis codes: non–AIDS-related infections, malignancy, liver disease, cardiovascular disease, respiratory disease, accident and suicide and overdose, and other. ARM, NARM, and all-cause mortality were calculated using the number of AIDS-related, non–AIDS-related, and all deaths and the corresponding duration of follow-up, respectively.

### Statistical Analysis

Descriptive statistics were used to summarize baseline demographic characteristics and HIV treatment outcomes for both the exposure and control groups. Differences between groups were compared using the Pearson chi-square test. Person-years (PY) was used to estimate ARM, NARM, and all-cause mortality. The Kaplan-Meier method was used to generate survival curves, and the log rank test was conducted to assess differences in survival time between the exposure and control groups. Competing risk models were used to identify factors potentially associated with ARM. Subdistribution hazard ratios were reported with corresponding 95% CIs. Cox proportional hazards models were used to assess correlates of all-cause mortality with reporting hazard ratios and its 95% CIs. Sex, age at diagnosis, ethnicity, marital status, education, occupation, the mode of transmission, the date of HIV diagnosis, baseline CD4 count, the receipt of peer-led community-based support, adherence to ART, and retention in care were included in the adjusted analysis for correlates of mortality and mortality, in which variables with a *P* value <.05 were retained in the final model. Statistical analyses were carried out using Stata (version 15.0; Stata Corp), and survival curves were completed using R software (version 3.6.0; R Core Team).

### Ethics Approval

This study was approved by the Ethics Review Committee of Wuxi Municipal Centre for Disease Control and Prevention (WXCDC2022014). A unique ID number was used to protect each participant’s privacy.

## Results

### Participant Characteristics

Data describing a total of 2794 people living with HIV were retrieved from the CNHCIMS between 2006 and 2019, among whom 430 met the criteria to be assigned to the exposure group. There were significant differences in baseline characteristics, including sex (*P*=.005), age at diagnosis (*P*<.001), education (*P*<.001), marital status (*P*<.001), occupation (*P*<.001), and baseline CD4 count (*P*=.001), comparing the 430 and 2364 people living with HIV who did and did not meet the inclusion criteria to be assigned to the exposure group, respectively. Using PSM, 430 people living with HIV were assigned to the control group. After PSM, people living with HIV in the exposure and control groups were comparable in all recorded baseline characteristics (Table 1).

**Table 1:** Baseline characteristics of people living with HIV assigned to the exposure and control groups before and after propensity score matching (PSM).

Variables			Before PSM								After PSM					
			Exposure group (n=430), n (%)		Other people living with HIV (n=2364), n (%)		*P* value			Exposure group (n=430), n (%)			Control group (n=430), n (%)		*P* value	
**Sex**								.005								.77
	Male	365 (84.9)		2117 (89.6)					365 (84.9)			368 (85.6)				
	Female	65 (15.1)		247 (10.5)					65 (15.1)			62 (14.4)				
**Age (years) at diagnosis**								<.001								.89
	<30	196 (45.6)		741 (31.4)					196 (45.6)			198 (46.1)				
	≥30	234 (54.4)		1623 (68.6)					234 (54.4)			232 (54)				
**Ethnicity**								.61								.73
	Han ethnicity	425 (98.8)		2329 (98.5)					425 (98.8)			426 (99.1)				
	Others	5 (1.2)		35 (1.5)					5 (1.2)			4 (0.9)				
**Education**								<.001								.27
	Primary or less	16 (3.7)		280 (11.8)					16 (3.7)			23 (5.4)				
	Secondary	234 (54.4)		1404 (59.4)					234 (54.4)			214 (49.7)				
	Tertiary	180 (41.9)		680 (28.8)					180 (41.9)			193 (44.9)				
**Marital status**								<.001								.09
	Unmarried	210 (48.8)		859 (36.3)					210 (48.8)			228 (53)				
	Married	147 (34.2)		790 (33.4)					147 (34.2)			118 (27.5)				
	Divorced and widowed	73 (17)		715 (30.3)					73 (17)			84 (19.5)				
**Occupation**								<.001								.90
	Student	41 (9.5)		69 (2.9)					41 (9.5)			42 (9.8)				
	Others	389 (90.5)		2295 (97.1)					389 (90.5)			388 (90.2)				
**Route of HIV infection**								.23								.94
	MSM^a^	262 (60.9)		1511 (63.9)					262 (60.9)			261 (60.7)				
	Others	168 (39.1)		853 (36.1)					168 (39.1)			169 (39.3)				
**Date of HIV diagnosis^b^**								.50								.62
	Before June 2016	275 (64)		1472 (62.3)					275 (64)			268 (62.3)				
	After June 2016	155 (36.1)		892 (37.7)					155 (36.1)			162 (37.7)				
**Baseline CD4 count (cells/μL)**								.001								.76
	<200	126 (29.3)		899 (38)					126 (29.3)			122 (28.4)				
	≥200	304 (70.7)		1465 (62)					304 (70.7)			308 (71.6)				

^a^MSM: men who have sex with men.

^b^Since June 2016, all people living with HIV in China have been encouraged to initiate antiretroviral therapy regardless of CD4 cell count.

Among all 860 people living with HIV included in our analysis, 733 (85.2%) were men, 394 (45.8%) were diagnosed with HIV aged <30 years, 851 (99%) were of Han ethnicity, 373 (43.4%) had completed high school or had a higher level of educational achievement, and 83 (9.7%) were current students. More than half were unmarried (438/860, 50.9%) and were men who have sex with men (523/860, 60.8%). More than one-third (317/860, 36.9%) were diagnosed after June 2016, and just under one-third (248/860, 28.8%) had a CD4 count <200 cells/μL at the time of HIV diagnosis.

### ART Adherence and Retention in Care

Compared with the control group, people living with HIV in the exposure group were more likely to adhere to ART (396/430, 92.1% vs 360/430, 83.7%; *χ*_1_^2^=14.2; *P*<.001) and remain retained in care (402/430, 93.5% vs 327/430, 76.1%; *χ*_1_^2^=50.7; *P*<.001) 12 months after ART initiation (Table 2). The proportion of people living with HIV who received viral load monitoring increased over time for both the exposure group (from 88/430, 20.5% at 9-12 months after HIV diagnosis to 381/430, 88.6% at 9-24 months after HIV diagnosis; *χ*_4_^2^=501.5; trend: *P*<.001) and control group (from 59/430, 13.7% to 243/430, 56.5%; *χ*_4_^2^=211.4; trend: *P*<.001; Table 3). Rates of viral load monitoring in the exposure group were significantly higher than the rates in the control group from 9 to 24 months after HIV diagnosis (all *P* values <.05). Rates of viral load suppression were significantly higher in the exposure group than the control group from 9 to 24 months after HIV diagnosis (357/381, 93.7% vs 217/243, 89.3%; *χ*_1_^2^=3.9; *P*=.048).

**Table 2:** Antiretroviral therapy (ART) adherence and retention in HIV care between the exposure and control groups (n=860).

Variables			Total (n=860), n (%)		Exposure group (n=430), n (%)		Control group (n=430), n (%)		Chi-square (*df*)			*P* value	
**Adhere to ART^a,b^**										14.2 (1)			<.001
	Yes	756 (87.9)		396 (92.1)		360 (83.7)							
	No	104 (12.1)		34 (7.9)		70 (16.3)							
**Retention in care^c^**										50.7 (1)			<.001
	Yes	729 (84.8)		402 (93.5)		327 (76.1)							
	No	131 (15.2)		28 (6.5)		103 (24)							

^a^ART: antiretroviral therapy.

^b^Adherence to ART was defined as never having a documented period of ART interruption and never having a period when a scheduled follow-up clinic visit was missed by more than 3 months.

^c^Retention in care was defined as receiving either CD4 or viral load monitoring within 12 months after ART initiation.

**Table 3:** Viral suppression between the exposure and control groups (n=860).

Variables		Exposure group						Control group				
		Values, n/N (%)	Chi-square (*df*)		Trend (*P* value)		Values, n (%)		Chi-square (*df*)		Trend (*P* value)	
**Viral load testing after enrollment (months)**				501.5 (4)		<.001				211.4 (4)		<.001
	9-12	88/430 (20.5)					59/430 (13.7)					
	9-15	211/430 (49.1)					115/430 (26.7)					
	9-18	292/430 (67.9)					169/430 (39.3)					
	9-21	341/430 (79.3)					207/430 (48.1)					
	9-24	381/430 (88.6)					243/430 (56.5)					
**Viral load suppression after enrollment (months)**				0.02 (4)		.89				0.1 (4)		.79
	9-12	83/88 (94.3)					52/59 (88.1)					
	9-15	196/211 (92.9)					105/115 (91.3)					
	9-18	271/292 (92.8)					154/169 (91.1)					
	9-21	317/341 (93)					187/207 (90.3)					
	9-24	357/381 (93.7)					217/243 (89.3)					

### ARM and All-Cause Mortality

During the study period, 25 individuals died within 4316.8 PY of follow-up. Among the 25 people living with HIV who died, 19 (76%) and 6 (24%) were attributed to ARM and NARM, respectively. Reasons for ARM were AIDS-related opportunistic infections (9/19, 47%), AIDS-related malignancies (6/19, 31%), and HIV wasting syndrome (4/19, 21%). Compared with the control group, the exposure group had lower rates of ARM (1.8 vs 7.0 per 1000 PY; rate difference −5.2; *P*=.01) and all-cause mortality (2.3 vs 9.3 per 1000 PY; rate difference −7.0; *P*=.002). There was no statistically significant difference in NARM (0.5 vs 2.3 per 1000 PY; rate difference −1.9; *P*=.12) comparing the 2 groups.

**Figure 2:**
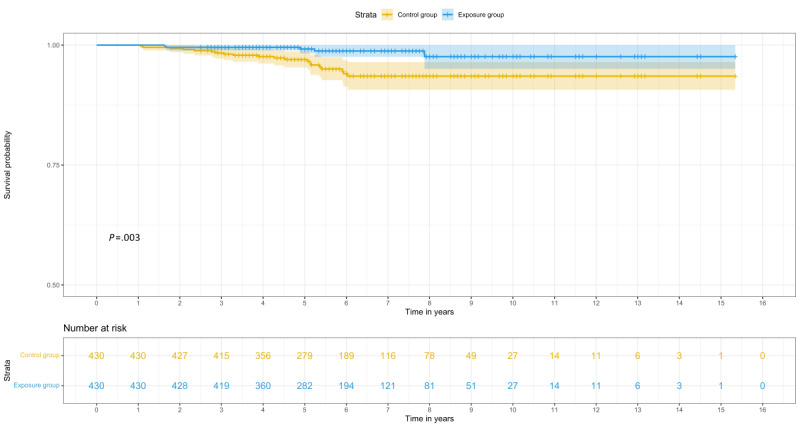
All-cause mortality between the exposure and control groups.

Survival analysis showed that people living with HIV in the exposure group had higher cumulative survival rates (*χ*_1_^2^=9.1; *P=*.003) compared with controls (Figure 2). After adjusting for other factors, multivariate analysis showed that the receipt of peer-led community-based support was associated with a 72% (adjusted subdistribution hazard ratio 0.28, 95% CI 0.09-0.95) reduction in ARM and a 70% (adjusted hazard ratio 0.30, 95% CI 0.11-0.82) reduction in all-cause mortality. Other factors associated with ARM and all-cause mortality are presented in Table 4.

**Table 4:** Correlates of AIDS-related mortality (ARM) and all-cause mortality among people living with HIV.

Characteristics		PY^a^ of follow-up	ARM				All-cause mortality		
			Deaths, n	Mortality rate (per 1000 PY; %)	Adjusted SHR^b^ (95% CI)	Deaths, n		Mortality rate (per 1000 PY; %)	Adjusted HR^c^ (95% CI)
**Sex**									
	Male	3650.43	17	4.7	Reference	22		6.0	Reference
	Female	666.47	2	3.0	0.32 (0.05-1.87)^d^	3		4.5	0.30 (0.08-1.12)^d^
**Age (years) at diagnosis**									
	<30	1934.85	4	2.1	Reference	4		2.1	Reference
	≥30	2382.05	15	6.3	1.99 (0.52-7.61)	21		8.8	2.44 (0.56-10.77)
**Ethnicity**									
	Han ethnicity	4206.67	18	4.3	Reference	24		5.7	Reference
	Others	110.23	1	9.1	1.46 (0.24-8.79)	1		9.1	1.25 (0.15-10.15)^d^
**Marital status**									
	Unmarried	2143.83	6	2.8	Reference	6		2.8	Reference
	Married	1424.30	6	4.2	0.67 (0.20-2.28)	10		7.0	1.08 (0.28-4.16)
	Divorced and widowed	748.77	7	9.3	1.10 (0.29-4.15)	9		12.0	1.43 (0.35-5.85)
**Education**									
	Primary or less	196.50	1	5.1	Reference	3		15.3	Reference
	Secondary	2320.90	14	6.0	1.85 (0.16-21.30)^d^	18		7.8	0.67 (0.18-2.47)
	Higher	1799.50	4	2.2	1.34 (0.07-25.00)^d^	4		2.2	0.36 (0.06-1.98)
**Occupation**									
	Student	397.19	1	2.5	Reference	1		2.5	Reference
	Others	3919.71	18	4.6	0.61 (0.07-5.76)^d^	24		6.1	0.76 (0.07-7.71)
**Mode of HIV transmission**									
	MSM^e^	2554.63	6	2.3	Reference	9		3.5	Reference
	Others	1762.27	13	7.4	3.17 (1.19-8.41)	16		9.1	1.82 (0.67-4.94)
**Date of HIV diagnosis**									
	Before June 2016	2997.84	15	5.0	Reference	20		6.7	Reference
	After June 2016	1319.06	4	3.0	0.69 (0.16-2.97)^d^	5		3.8	0.43 (0.14-1.30)^d^
**Baseline CD4 count (cells/μL)**									
	<200	1290.49	13	10.1	Reference	15		11.6	Reference
	≥200	3026.40	6	2.0	0.24 (0.09-0.66)	10		3.3	0.39 (0.17-0.90)
**Receipt of peer-led community-based support**									
	No	2144.74	15	7.0	Reference	20		9.3	Reference
	Yes	2172.12	4	1.8	0.28 (0.09-0.95)	5		2.3	0.30 (0.11-0.82)
**Adherence to ART^f^**									
	Yes	3760.96	10	2.7	Reference	15		4.0	Reference
	No	555.94	9	16.2	4.55 (1.74-12.50)	10		18.0	3.70 (1.56-8.33)
**Retention in care**									
	No	855.34	6	7.0	Reference	10		11.7	Reference
	Yes	3461.56	13	3.8	0.46 (0.14-1.54)	15		4.3	0.27 (0.11-0.64)

^a^PY: person-years.

^b^SHR: subdistribution hazard ratio.

^c^HR: hazard ratio.

^d^These variables were tested associated with mortality at *P*>.20, which were not included in the initial adjusted model.

^e^MSM: men who have sex with men.

^f^ART: antiretroviral therapy.

## Discussion

### Principal Findings

In this propensity score–matched analysis of HIV treatment outcomes and mortality among people living with HIV in Wuxi between 2006 and 2021, we found that people living with HIV who received peer-led community-based support had better ART adherence, higher rates of retention in care, and improved survival rates than people living with HIV who had not received these services. Rates of viral suppression and compliance to viral load monitoring guidelines were high in the exposure group, with >90% of people living with HIV who had participated in the multicomponent support services program achieving viral suppression 9 to 24 months after HIV diagnosis. Most previous studies evaluating the relationship between peer-led support and HIV treatment outcomes were implemented in low- to middle-income countries (LMICs) in Africa or high-income countries, with few previous studies having been conducted in Asia [27,28]. Previous evaluations of similar interventions also reported improvements in ART adherence, viral suppression, and retention in care, although few have evaluated associations with ARM or all-cause mortality, given the limited follow-up periods [15,29]. Our study adds to the existing literature through demonstrating associations between peer-led community-based support services and improved HIV treatment outcomes, including lower ARM and all-cause mortality, over 15 years of follow-up in a middle-income country in Asia.

### The Impact of Peer Support on Improving ART Adherence, Retention in Care, and Viral Suppression

People living with HIV in China who received at least 6 months of adjunctive peer-led community-based support had better ART adherence, viral suppression, and retention in HIV care than those in matched controls who only received standard clinic-based HIV care. Differences in retention in care were particularly pronounced, with >93% and only 76% of people living with HIV in the exposure and control groups retained in HIV care for ≥12 months after ART initiation, respectively. Our findings are similar to previous observational and experimental evaluations of support programs delivered by CHWs to people living with HIV in LMICs [15]. A prospective observational cohort study in Rwanda found that 85% of people living with HIV who received supplementary community-based support services remained retained in care and virally suppressed 1 year after ART initiation, which was a significantly higher proportion than people living with HIV who only received standard clinic-based HIV care (79%) [17]. Several meta-analyses have shown that community-based HIV initiatives and peer support are superior to standard clinic-based care to improve retention in care, ART adherence, and viral suppression [30,31]. There are many possible explanations as to why the receipt of peer-led community-based support was associated with improved HIV treatment outcomes in our analysis. The fear of unintended disclosure of HIV status, stigma, and discrimination have been identified as negatively influencing ART adherence and retention in care [32,33]. The SMART support framework used by CHWs at Rainbow Family explicitly addresses these potential barriers. It includes social and emotional support to help people living with HIV cope with stresses associated with partner notification and the disclosure of HIV status, as well as assistance navigating stigma and discrimination from health care providers. Previous studies have also found anxiety, depression, and treatment fatigue as negatively impacting ART adherence and retention in care [34,35]. The psychological support provided by CHWs in this study included specific strategies to address concurrent anxiety and depression among people living with HIV. CHWs at Rainbow Family also provided reminders regarding medication refills and laboratory testing in efforts to mitigate treatment fatigue, which may have improved rates of ART adherence and viral load monitoring among people living with HIV in the exposure group.

### The Impact of Peer Support on Reductions of ARM and All-Cause Mortality

Compared with matched controls, the receipt of peer-led community-based support services was associated with 72% and 70% reductions in ARM and all-cause mortality, respectively, among people living with HIV in China. These findings are consistent with previous studies conducted in Rwanda [17] and South Africa [36] where support services delivered by CHWs in community settings were associated with lower mortality among people living with HIV after ART initiation. The improved HIV treatment outcomes observed among people living with HIV who received supportive services from Rainbow Family were likely a major contributing factor to the higher cumulative survival in the exposure group. Decades of research have shown that retention in care and ART adherence are essential to achieving and maintaining viral suppression, improving immune function, and thereby reducing ARM among people living with HIV [37-42]. Our results suggest that the observed reductions in all-cause mortality between the exposure and control groups were primarily driven by reductions in ARM as NARM did not significantly differ between the 2 groups. Although CHWs at Rainbow Family did provide general education on healthy lifestyle choices beyond HIV care, including the importance of physical activity, balanced diet, and smoking cessation, these were secondary considerations and were not explicitly emphasized by any particular component of the SMART support framework. As the proportion of deaths attributable to NARM among people living with HIV continues to grow worldwide, the scope of CHW support for people living with HIV may need to be expanded to address causes of mortality beyond opportunistic infections and HIV- or AIDS-associated malignancies, such as cardiovascular disease, substance use disorder, renal disease, and liver disease [13].

### Differences in Various Peer-Led Community-Based Support Models

The peer-led community-based support services provided by Rainbow Family adopted a multicomponent SMART framework that attempted to provide broad support for HIV care across multiple domains, including HIV education and counseling, ART adherence and laboratory monitoring reminders, social and emotional support for interpersonal relationships and disclosure of HIV status, and mental health counseling. Although the WHO guidelines encourage task shifting of HIV services to CHWs in resource-limited settings, little guidance is available as to which types of support services or delivery models should be prioritized in task-shifting efforts to optimize health outcomes among people living with HIV [14]. Systemic reviews of CHWs interventions for people living with HIV in LMICs have found that support services vary considerably. Many CHW-based or peer-led interventions described in the literature include regular phone calls, SMS text messages, or home visits to answer HIV care questions and reminders to obtain ART refills and laboratory monitoring [31]. Other components that have been inconsistently incorporated into CHW-based or peer-led interventions for people living with HIV include directly observed therapy, referrals to other medical or social work services, harm reduction and substance use management, and various forms of psychosocial support [31]. The wide variety of intervention models as well as substantial differences in study design, setting, and measured outcomes complicate efforts to perform comparative analyses to determine which forms of peer-led community-based support are the most important to improve HIV treatment outcomes among people living with HIV [27]. Future qualitative and implementation science work may be particularly helpful in identifying core components of peer-led community-based support services for people living with HIV and optimizing service delivery across different settings in LMICs.

### Engaging CBOs in HIV Care in China

Although the Chinese government has established the China AIDS Fund to support the development of grassroots organizations including CBOs providing service to people living with HIV, there are only a small number of people living with HIV who are served by peer CHWs. As CBOs in China are required to register as an official nongovernmental organization in a local Civil Affairs Bureau and most of them do not have sufficient personnel and funding to support themselves to achieve this, they have to apply for governmental funding on HIV as grassroots organizations through cooperation with local CDCs or specialized infectious disease hospital, which limits their development and growth owing to lacking endorsement from the government [43,44]. Our study showed that providing peer-led community-based differentiated support through peer CHWs in CBOs were likely to significantly improve the survival of people living with HIV. Accordingly, we strongly suggest that the central government and local governments at all levels should formulate more supportive policies and invest more to engage CBOs in HIV care. Because people living with HIV in China are generally experiencing a high degree of stigma owing to the conservative social environment [45], many infected persons are not willing to join CBOs to work as a peer CHW. Consequently, multilateral and multilevel efforts are urgently needed to overcome stigma and discrimination against people living with HIV to encourage them to engage in providing peer-led community-based differentiated support services.

### Comparison With Prior Work

To the best of our knowledge, this is the first study to evaluate associations between the receipt of a multicomponent peer-led support service and survival among people living with HIV over a 15-year follow-up period and in a country in Asia based on a propensity score–matched analysis.

### Limitations

Our study has several important limitations. The nonrandomized retrospective nature of our analysis prevents us from determining whether peer-led community-based support caused the observed differences in HIV treatment outcomes and survival between the exposure and control groups. Confounding factors may be responsible for some or all of our observed associations. For example, it is possible people living with HIV who were experiencing a mental health crisis or socioeconomic instability were both systematically more likely to have poor HIV treatment outcomes and less likely to remain connected to support services at Rainbow Family for at least 6 months. We used PSM to match individuals in the exposure and control groups to control for many common confounders that are known to influence HIV treatment outcomes; however, the possibility of unmeasured confounders influencing our results remains. Of note, by limiting our analytical sample to the 430 people living with HIV who received peer-led community-based support and 430 of matched controls, the representativeness of our sample may have been impacted, and people living with HIV included in this analysis may not be representative of people living with HIV in China more broadly. Therefore, the generalizability of our results must be interpreted with caution. In addition, although Rainbow Family was the only CBO registered with the local government to provide community-based support services to people living with HIV in Wuxi between 2006 and 2021, it is possible that other local CBOs or nongovernmental organizations were providing support services to some of the persons included in our analysis that were unknown to the local government or these authors, resulting in misclassification bias.

### Conclusions

This study adds additional evidence supporting the use of peer-led community-based HIV initiatives and peer support interventions in LMICs, demonstrating that these programs are likely impactful in resource-limited settings beyond sub-Saharan Africa. The SMART framework used to guide the multicomponent HIV support services provided by Rainbow Family may be a model for similar services in other LMICs, particularly in other parts of China and East Asia where comprehensive peer-led support for people living with HIV remains less common. Further studies conducted across diverse settings are needed to confirm the effect and cost-effectiveness of such interventions.

## Abbreviations

ARM: AIDS-related mortality
ART: antiretroviral therapy
CBO: community-based organization
CDC: center for disease control and prevention
CHW: community health worker
CNHCIMS: Chinese National HIV/AIDS Comprehensive Information Management System
LMIC: low- to middle-income country
NARM: non–AIDS-related mortality
PSM: propensity score matching
PY: person-years
SMART: social, medical, and mental antiretroviral therapy support
WHO: World Health Organization
